# The Three Faces of IgA Nephropathy: Clinicopathological Heterogeneity and Urinary Albumin-to-Protein Ratio in a Case Series

**DOI:** 10.3390/jcm15176646

**Published:** 2026-08-28

**Authors:** Anna Masajtis-Zagajewska, Blazej Kieszek, Michał Nowicki

**Affiliations:** Department of Nephrology, Hypertension, Transplantation and Internal Medicine, Central University Hospital, Medical University of Lodz, 90-419 Lodz, Poland; blazej.kieszek@umed.lodz.pl (B.K.); michal.nowicki@umed.lodz.pl (M.N.)

**Keywords:** IgA nephropathy, urinary protein composition, uAPR, podocytopathy, FSGS, electron microscopy

## Abstract

**Background/Objectives:** IgA nephropathy (IgAN) is a highly heterogeneous glomerular disease with variable clinical presentation and response to therapy. Standard histopathology provides a static assessment and may not fully reflect dynamic podocyte injury. The objective of this case series was to characterize distinct clinical phenotypes of IgAN and explore the relationship between urinary protein composition assessed by the urine albumin-to-protein ratio (uAPR) and ultrastructural glomerular injury. **Methods:** We retrospectively analyzed three patients with distinct clinical phenotypes of biopsy-proven IgA nephropathy to examine the relationship between urinary protein composition (uAPR), ultrastructural findings, treatment response, and clinical outcomes. **Results:** Case 1 demonstrated a podocytopathic phenotype resembling minimal change disease (MCD) with steroid sensitivity. Case 2 showed progression to focal segmental glomerulosclerosis (FSGS) despite stable renal function. Case 3 revealed persistent predominantly non-albumin proteinuria (uAPR 0.07–0.13) preceding biopsy-proven IgAN and resistance to multiple immunosuppressive regimens. **Conclusions:** Differences in uAPR were observed alongside distinct clinicopathological and ultrastructural phenotypes of IgA nephropathy. These observations suggest that urinary protein composition may provide complementary information on disease phenotype; however, its relationship with specific mechanisms of glomerular injury requires prospective validation.

## 1. Introduction

IgA nephropathy (IgAN) is a highly heterogeneous glomerular disease with variable clinical presentation, histopathological features, and therapeutic response. Although its pathogenesis is driven by mesangial deposition of galactose-deficient IgA1 (Gd-IgA1)-containing immune complexes [1], the clinical course is largely determined by the extent and nature of secondary injury to the glomerular filtration barrier. Clinical presentation generally correlates with the severity of histopathological injury [2,3,4]. Among all IgAN subtypes, cases presenting with nephrotic syndrome and mild mesangial proliferation are relatively rare, accounting for approximately 5–10% of all IgAN patients [5]. Therefore, this variant of IgAN, characterized by nephrotic syndrome and diffuse podocyte foot process effacement on electron microscopy (EM) resembling minimal change disease (MCD), is defined as MCD-IgAN [2].

In general, a correlation exists between the clinical presentation and pathological features of IgAN. Increasing evidence suggests that severe proteinuria at diagnosis is associated with a more aggressive disease course, accompanied by significant glomerular injury or renal insufficiency [6]. Conversely, other studies have described clinical features of a podocytopathic variant with a favorable response to glucocorticoid therapy in patients with MCD-IgAN, resembling the response observed in patients with MCD [7,8,9]. Therefore, it remains controversial whether these patients represent a distinct podocytopathic variant of IgAN, a coexistence of MCD with mild IgAN, or MCD with non-pathogenic IgA deposition [3,10].

Modern nephrology faces the challenge of precise stratification of these patients. Although light microscopy often reveals only minimal mesangial changes, the actual fate of nephrons depends on the ultrastructural integrity of the glomerular filtration barrier [11]. Electron microscopy remains the gold standard for this assessment, allowing visualization of podocyte foot process effacement, podocyte depletion, and alterations in the glomerular basement membrane [12]. Classical histopathological evaluation based on light microscopy and the Oxford classification (MEST-C) [13], together with clinical assessment of proteinuria and kidney function as recommended by current KDIGO guidelines [14], remains central to the evaluation and risk stratification of patients with IgAN. However, histopathology represents a static “snapshot” of renal structure, which does not always correlate with the dynamic process of podocyte injury, particularly in centers lacking routine access to electron microscopy. Despite its diagnostic value, kidney biopsy with EM assessment is an invasive, costly procedure and is difficult to repeat serially for monitoring disease dynamics [12]. In the era of precision medicine, there is an increasing demand for non-invasive tools that could provide complementary information on disease phenotype and evolution between kidney biopsies.

The inability to assess ultrastructural features such as podocyte foot process effacement or early stages of Focal Segmental Glomerulosclerosis (FSGS) creates a diagnostic gap that may lead to suboptimal therapeutic decisions—ranging from unnecessary and potentially toxic immunosuppression to delayed initiation of aggressive nephroprotective strategies.

In response to these challenges, there is growing interest in simple, non-invasive parameters that may provide complementary information on urinary protein composition and kidney disease phenotype. One such approach is assessment of urinary protein composition using the urinary albumin-to-creatinine ratio (uACR) and urinary protein-to-creatinine ratio (uPCR).

The urine albumin-to-protein ratio (uAPR = uACR/uPCR) provides a simple quantitative measure of the relative contribution of albumin to total urinary protein and has been investigated as a marker of urinary protein composition and kidney disease phenotype [15,16,17].

The concept of protein selectivity was first described in 1964 as a predictor of steroid responsiveness in adult nephrotic syndrome [18]. Subsequently, its prognostic value has also been investigated in other glomerular diseases, including as a predictor of treatment response and renal outcomes in lupus nephritis [19]. Urinary protein electrophoresis (UPE), another method for evaluating proteinuria (typically in 24 h urine collections), provides qualitative information regarding urinary protein composition and protein origin [15,20]. However, it has been hypothesized that the type of proteinuria, as determined by electrophoretic patterns and immunofixation, may be predicted using a simple ratio of low-molecular-weight proteins, such as albumin, to total urinary protein, i.e., the APR index [15].

Although no universal cut-off values for uAPR have been established, uAPR should be interpreted as a continuous variable, with increasing values reflecting a greater proportion of albumin within total urinary protein and lower values indicating a relatively greater contribution of non-albumin proteins.

**Figure 1:**
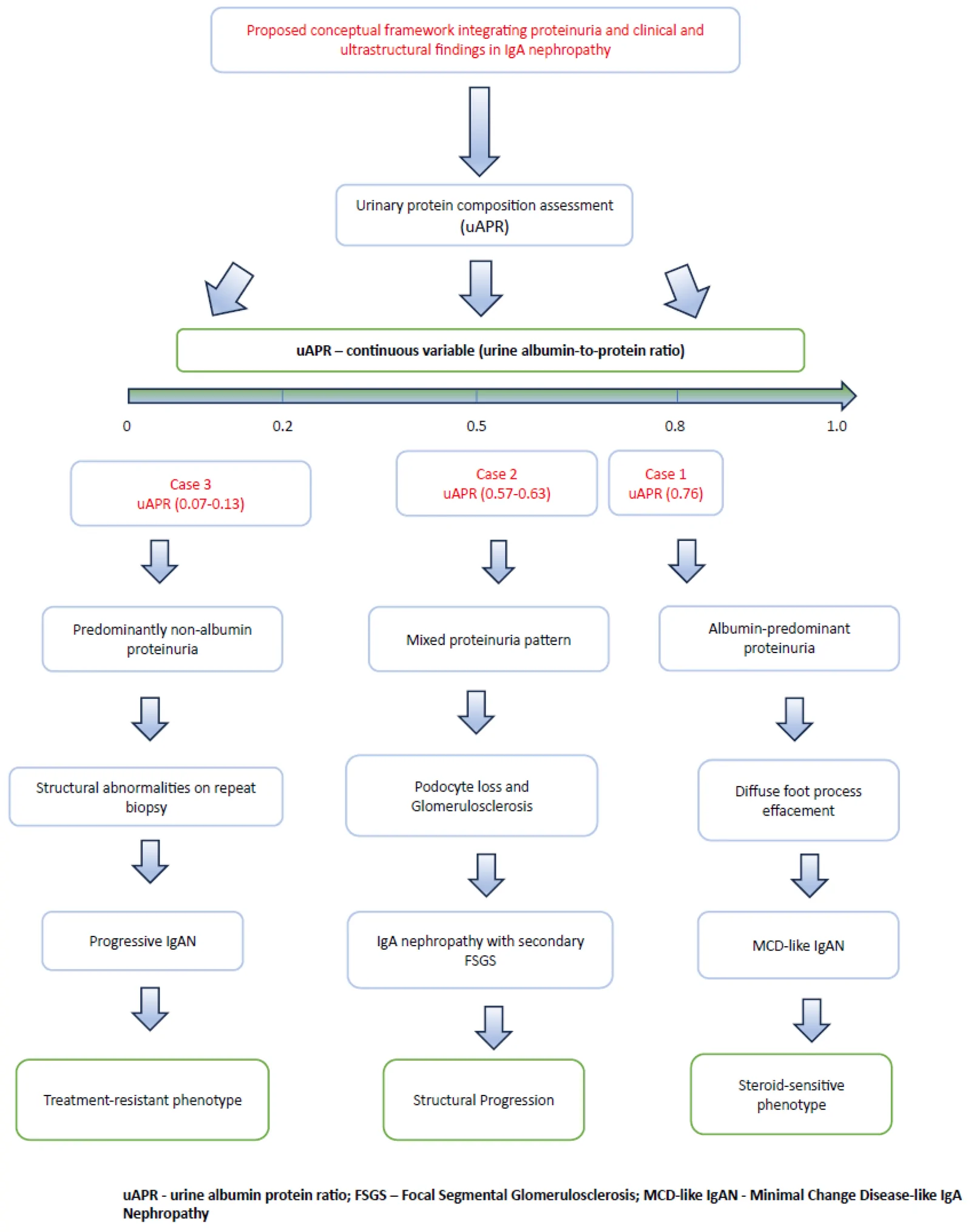
Proposed conceptual framework integrating urinary protein composition assessed by urine albumin-to-protein ratio (uAPR) with the clinical, histopathological, and ultrastructural findings observed in the three presented cases. uAPR is presented as a continuous variable reflecting the relative contribution of albumin to total urinary protein. The three cases illustrate different urinary protein composition profiles occurring alongside distinct clinicopathological and ultrastructural phenotypes. The relationships shown are hypothesis-generating and represent the authors’ interpretation of these observations; they should not be interpreted as evidence of causality or as validated diagnostic or prognostic uAPR thresholds.

We hypothesized that distinct clinical phenotypes of IgA nephropathy may be associated with different patterns of urinary protein composition and ultrastructural injury. The proposed conceptual framework is illustrated in Figure 1.

The aim of this study was to describe distinct clinical phenotypes of IgA nephropathy and explore the relationship between urinary protein composition (uAPR), patterns of glomerular injury, and ultrastructural findings. Particular attention was given to the relationship between uAPR profiles, treatment response, and disease course across diverse clinical scenarios.

The mechanistic interpretation presented throughout this manuscript should be regarded as a hypothesis-generating conceptual framework derived from the integration of clinical, histopathological, ultrastructural and proteinuria data rather than as direct evidence of causality.

## 2. Materials and Methods

### 2.1. Study Design

This retrospective descriptive case series included three patients with biopsy-proven IgA nephropathy representing distinct clinicopathological phenotypes. Cases were selected to illustrate distinct clinicopathological phenotypes of IgA nephropathy rather than to represent the full clinical spectrum of the disease. Clinical presentation, laboratory findings, histopathological evaluation, electron microscopy, treatment response, and longitudinal outcomes were analyzed to explore the relationship between urinary protein composition (uAPR) and ultrastructural patterns of glomerular injury.

### 2.2. Case Selection

This case series was designed to illustrate three distinct clinicopathological phenotypes of IgA nephropathy rather than to represent the full clinical spectrum of the disease. Cases were purposively selected from patients managed at our center based on the availability of:

- Biopsy-confirmed IgA nephropathy,
- Complete clinical, laboratory and electron microscopy data,
- Longitudinal follow-up,
- Assessment of urinary protein composition (uAPR), including paired measurements at biopsy time points where available,
- Repeat kidney biopsy allowing evaluation of structural disease evolution whenever available.

Because repeat kidney biopsy is rarely performed in routine clinical practice in patients with IgA nephropathy, cases with repeat histopathological evaluation provided a unique opportunity to correlate longitudinal changes in urinary protein composition with ultrastructural and clinicopathological evolution.

The selected cases were considered representative of three contrasting clinical scenarios encountered in IgA nephropathy: (1) a steroid-responsive podocytopathic phenotype (MCD-like IgAN), (2) progressive structural remodeling toward secondary FSGS despite stable kidney function, and (3) treatment-resistant disease with early predominantly non-albumin proteinuria preceding overt clinicopathological progression.

### 2.3. Proteinuria Assessment

Urinary albumin and total protein concentrations were measured using immunoturbidimetric and colorimetric methods, respectively. uACR and uPCR were determined simultaneously in the same first-morning urine sample, and uAPR was calculated as uACR/uPCR. Baseline samples were collected before the initial kidney biopsy, whereas follow-up samples in Cases 2 and 3 were collected before the repeat kidney biopsy. Urinary albumin-to-creatinine ratio (uACR) and urinary protein-to-creatinine ratio (uPCR) were measured as part of routine clinical assessment. The urinary albumin-to-protein ratio (uAPR) was calculated as the ratio of uACR to uPCR. uAPR represents the proportion of total urinary protein accounted for by albumin and should not be considered equivalent to the conventional proteinuria selectivity index based on the relative clearance of proteins of different molecular weights. Higher uAPR values indicate a greater relative contribution of albumin to total urinary protein, whereas lower values indicate a greater contribution of non-albumin proteins. Low uAPR values are not specific for glomerular structural injury and may also occur in tubular or other forms of predominantly non-albumin proteinuria. uAPR was therefore analyzed descriptively as a continuous variable and explored in relation to histopathological findings, ultrastructural features, treatment response, and longitudinal clinical outcomes.

### 2.4. Histopathological Assessment

Kidney biopsy specimens were evaluated by light microscopy, immunofluorescence, and electron microscopy according to standard routine diagnostic practice. Histopathological lesions of IgA nephropathy were classified using the Oxford MEST-C classification where applicable. Ultrastructural evaluation focused on the degree of podocyte foot process effacement, glomerular basement membrane alterations, mesangial expansion, and the presence of electron-dense immune deposits. Histopathological findings were interpreted in conjunction with the clinical presentation and urinary protein composition.

### 2.5. Ethical Considerations

According to institutional regulations, ethical review and approval were waived because this study represents a retrospective analysis of fully anonymized clinical data collected during routine medical care. The study was conducted in accordance with the principles of the Declaration of Helsinki. Written informed consent for the anonymous use of clinical data and publication of this case series was obtained from all patients.

### 2.6. Statistical Analysis

Because of the descriptive and hypothesis-generating nature of this three-case series, no formal statistical analyses were performed.

### 2.7. AI Statement

During the preparation of this manuscript, the authors used OpenAI ChatGPT (GPT-5) solely for language editing and improvement of textual clarity and readability. The tool was not used to generate or analyze data, produce results, or formulate scientific conclusions. The authors reviewed and edited all AI-assisted text and take full responsibility for the content of the manuscript.

## 3. Results

### 3.1. Case 1

A 32-year-old female with no significant past medical history presented with several days of massive, generalized edema. She denied fever, arthralgia, skin lesions, recent travel to endemic regions, medication use, and had no relevant family history. The onset of symptoms was preceded by a COVID-19 infection.

Laboratory findings at admission are summarized in Table 1. Given the presence of nephrotic-range proteinuria, an extensive diagnostic workup was performed to determine the etiology of the nephrotic syndrome. Renal function parameters were within normal limits. Laboratory evaluation revealed markedly elevated cholesterol levels and severe hypoalbuminemia. Complement components (C3, C4) were within normal range. Serum protein electrophoresis demonstrated profound hypoproteinemia with hypoalbuminemia. Antinuclear antibodies (ANAs), antineutrophil cytoplasmic antibodies (ANCAs), and anti-glomerular basement membrane (anti-GBM) antibodies were negative. Antistreptolysin O (ASO) titers were within normal limits. Serological tests for HIV and hepatotropic viruses were negative. Imaging studies showed no significant abnormalities.

Diuretic therapy was initiated, and a kidney biopsy was performed. Histopathological examination revealed IgA nephropathy, characterized by mesangial hypercellularity and granular mesangial staining for IgA (+3), C3 (+2), kappa light chains (+2), and lambda light chains (+3). No staining was observed for IgG, IgM, fibrinogen, or C1q. Electron microscopy demonstrated mesangial proliferation, expansion of the mesangial matrix, and electron-dense deposits within the mesangium corresponding to immune complexes. There was diffuse effacement of podocyte foot processes, while the glomerular basement membrane showed no ultrastructural abnormalities.

Glucocorticoid therapy was initiated, resulting in complete remission within a few days. However, attempts to taper prednisone were associated with rapid relapse of nephrotic syndrome, necessitating repeated courses of high-dose corticosteroids. Given the diagnosis of IgA nephropathy with steroid-dependent nephrotic syndrome, mycophenolate mofetil was added to the treatment regimen. Despite this, relapses continued to occur when the prednisone dose was reduced to 10 mg daily. Disease exacerbations were consistently triggered by infections.

Subsequently, a calcineurin inhibitor was introduced in place of mycophenolate mofetil. Cyclosporine was administered at doses maintaining trough levels of 100–150 ng/mL during the first three months, followed by 50–100 ng/mL thereafter. This regimen allowed reduction in prednisone to 5 mg daily while maintaining complete remission, despite intermittent infections.

### 3.2. Case 2

A 57-year-old female with a history of IgA nephropathy diagnosed several years earlier was followed under nephrological care. The initial kidney biopsy demonstrated approximately 10% globally sclerosed glomeruli. The remaining glomeruli showed mesangial hypercellularity with mild expansion of the mesangial matrix. Immunofluorescence revealed granular mesangial staining for IgA (+3), C3 (+2), kappa light chains (+1), and lambda light chains (+1), with no staining for IgG, IgM, C1q, or fibrinogen. Electron microscopy confirmed the presence of electron-dense mesangial deposits corresponding to immune complexes. Up to four mesangial cells per mesangial area were observed. Endothelial cells appeared swollen, while the glomerular basement membrane and podocytes showed no ultrastructural abnormalities.

The patient had persistent proteinuria of approximately 2.5 g/day for several years and was treated with nephroprotective therapy, including renin–angiotensin–aldosterone system (RAAS) blockade. She had previously received glucocorticoid therapy. In the months preceding reassessment, an increase in proteinuria to over 3 g/day was observed.

The patient was enrolled in a clinical trial evaluating the efficacy and safety of atacicept in patients with IgA nephropathy, which required a repeat kidney biopsy. Histopathological evaluation revealed features of FSGS, with approximately 25% of glomeruli globally sclerosed and segmental sclerosis present in 50% of the remaining glomeruli. Immunofluorescence was negative. Electron microscopy demonstrated expansion of the mesangial matrix without electron-dense immune deposits. The glomerular basement membrane was thickened and irregularly folded, and diffuse podocyte foot process effacement was observed.

Nephroprotective therapy was intensified. Throughout the observation period, estimated glomerular filtration rate (eGFR) remained stable despite histological progression.

The evolution toward FSGS observed in Case 2 is consistent with the broader concept that podocytopathic lesions may evolve along an MCD–FSGS spectrum in immune-mediated nephrotic disease [21].

Baseline and follow-up laboratory findings are summarized in Table 1.

### 3.3. Case 3

A 24-year-old male presented with sudden-onset nephrotic syndrome, with baseline proteinuria (uPCR) of 9600 mg/g and hypoalbuminemia of 33.2 g/L. Initial kidney biopsy evaluated by light microscopy showed minimal glomerular changes, while immunofluorescence was negative, leading to a preliminary diagnosis of MCD.

Assessment of urinary protein composition revealed a uAPR (uACR/uPCR) of 0.07, indicating that albumin accounted for only a small proportion of total urinary protein. This predominantly non-albumin proteinuria pattern was notable in the context of nephrotic-range proteinuria despite relatively unremarkable light microscopy findings.

The patient was initially treated with high-dose oral corticosteroids (1 mg/kg/day) but failed to achieve remission after eight weeks. Subsequent therapy with cyclosporine also did not result in a reduction in proteinuria. Due to the lack of therapeutic response, rituximab was administered; however, despite effective B-cell depletion, no clinical improvement was observed.

Over approximately two years of follow-up, kidney function progressively deteriorated, with eGFR declining from 98.1 to 43.2 mL/min/1.73 m^2^ and serum creatinine increasing from 96.1 to 187.3 μmol/L. This prompted a repeat kidney biopsy, which demonstrated features consistent with IgA nephropathy. Light microscopy revealed mesangial hypercellularity with expansion of the mesangial matrix, accompanied by segmental glomerulosclerosis and focal thickening of capillary walls. Immunofluorescence showed granular mesangial deposits of IgA (+2) and C3 (+2), with weaker staining for kappa (+1) and lambda (+1) light chains. No staining was detected for IgG, IgM, C1q, or fibrinogen.

Electron microscopy identified sparse electron-dense mesangial deposits corresponding to immune complexes, along with diffuse effacement of podocyte foot processes, indicating significant podocyte injury.

The patient was subsequently treated with targeted-release budesonide (9 mg/day) to address the gut–kidney axis. After six months of therapy, no significant reduction in proteinuria was observed. Systemic inflammatory markers remained within normal limits throughout the course.

At the time of repeat kidney biopsy, uAPR was 0.13, consistent with a predominantly non-albumin proteinuria pattern. Treatment was subsequently shifted toward maximal nephroprotective management.

**Figure 2:**
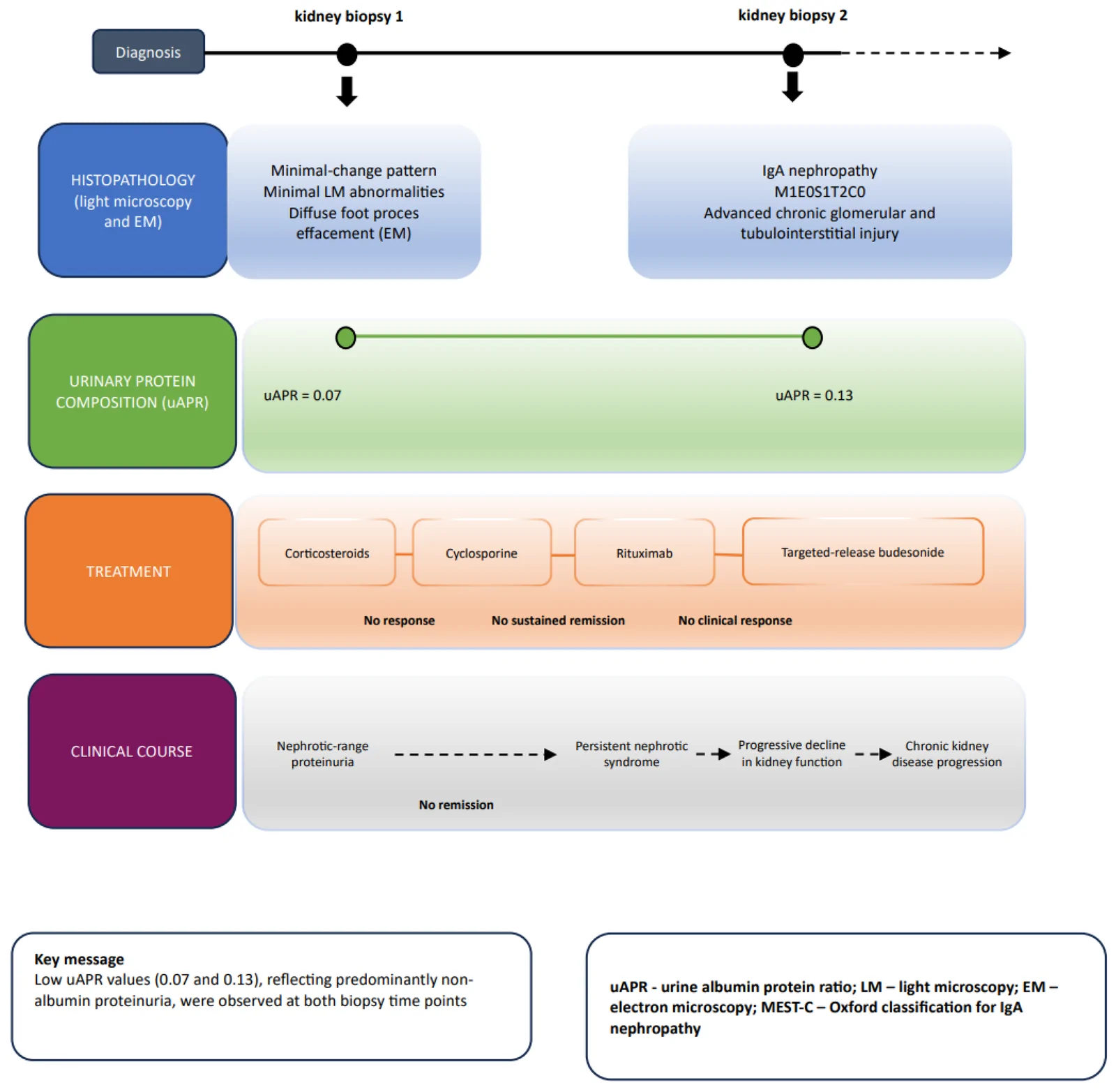
Longitudinal course of Case 3. Early predominantly non-albumin proteinuria, lack of response to successive therapies, kidney function decline, and repeat biopsy.

This case illustrates a predominantly non-albumin proteinuria pattern that was present early in the disease course and preceded both progressive loss of kidney function and the subsequent demonstration of more advanced structural abnormalities on repeat kidney biopsy. However, in the absence of direct urinary protein fractionation, the composition and origin of the non-albumin protein fraction cannot be determined. Electron microscopy at repeat biopsy demonstrated extensive podocyte injury. Low uAPR values were observed at both biopsy time points (0.07 and 0.13), while the clinical course was characterized by resistance to multiple lines of immunosuppressive therapy. The temporal relationship between uAPR measurements, therapeutic interventions, histopathological findings, and disease progression is summarized in Figure 2.

Baseline and follow-up laboratory findings are summarized in Table 1. Table 2 presents a comparison of three cases of IgA nephropathy.

Table 2 summarizes the principal clinicopathological differences among the three patients.

## 4. Discussion

The present study highlights the marked heterogeneity of IgA nephropathy and underscores the limitations of static histopathological assessment. Our observations support the concept that IgAN should be viewed not as a single disease entity, but rather as a spectrum of disorders involving distinct and potentially shifting pathophysiological mechanisms.

To our knowledge, this is the first study to explore the relationship between uAPR and ultrastructural findings across distinct clinical phenotypes of IgA nephropathy.

The first case represents a podocytopathic form of IgAN resembling Minimal Change Disease (MCD), commonly referred to as “IgA podocytopathy.” In this phenotype, podocyte dysfunction is the predominant mechanism of injury, resulting in albumin-predominant proteinuria and a clinical presentation of nephrotic syndrome. A favorable response to immunosuppressive therapy is characteristic and further aligns this variant with classical MCD. However, the presence of mesangial IgA deposits indicates a distinct initiating mechanism, while the podocytopathic features represent a functional manifestation of the disease.

The second case illustrates the progression of IgAN to Focal Segmental Glomerulosclerosis (FSGS). This transition can be interpreted as the endpoint of chronic glomerular injury, characterized by podocyte loss and secondary scarring. Notably, despite relative stability in clinical parameters such as serum creatinine and proteinuria, histological progression was evident. This observation underscores the limitations of relying solely on laboratory markers and highlights the importance of recognizing ongoing structural changes.

The third case is the most complex. The initial diagnosis of MCD in a patient presenting with nephrotic syndrome was challenged by the presence of predominantly non-albumin proteinuria, which is atypical for classical MCD. The lack of response to immunosuppressive therapy further questioned the initial diagnosis. Over time, deterioration of renal function and subsequent biopsy revealed features consistent with IgA nephropathy. The presence of a predominantly non-albumin proteinuria profile at both assessed biopsy time points was notable, with this pattern already present at initial presentation before the subsequent demonstration of more advanced structural abnormalities on repeat biopsy. One possible explanation is that IgA-related pathology may already have been present initially but was not captured in the first biopsy because of sampling limitations.

An observation emerging from these cases is that differences in urinary protein composition assessed by uAPR occurred alongside distinct ultrastructural and clinical phenotypes.

Based on these observations, we propose a conceptual model linking urinary protein composition, ultrastructural injury, and clinical phenotype in IgA nephropathy (Figure 1). Within this exploratory framework, uAPR is interpreted as a continuous descriptor of urinary protein composition rather than as a conventional proteinuria selectivity index. Higher values indicate a greater relative contribution of albumin to total urinary protein, whereas lower values indicate a greater contribution of non-albumin proteins. In the present cases, these differences in urinary protein composition occurred alongside distinct ultrastructural and clinical phenotypes. Whether such patterns consistently reflect specific mechanisms or severity of glomerular injury requires validation in larger prospective studies with direct characterization of urinary protein fractions. The observations from Case 2 further illustrate that relatively stable uAPR values may coexist with progressive structural remodeling and evolution toward secondary FSGS.

Previous studies have shown that the urine albumin-to-protein ratio may provide information on clinicopathological patterns and may help distinguish predominantly glomerular from non-glomerular proteinuria patterns [22]. Our observations extend this concept by raising the hypothesis that, when interpreted as a continuous variable, uAPR may also provide insight into different mechanisms of glomerular injury that may appear similar on light microscopy.

In the first case (reversible phenotype), a uAPR of 0.76 corresponded to electron microscopy findings showing diffuse podocyte foot process effacement without glomerular basement membrane disruption. This observation raises the hypothesis that higher uAPR values, interpreted as a continuous variable, may be associated with a predominantly functional podocytopathy and favorable responsiveness to corticosteroid therapy, resembling the phenotype of classical MCD.

In contrast, the third case (aggressive phenotype) demonstrated a markedly different profile.

uAPR values were low at both assessed biopsy time points (0.07 and 0.13) and occurred in association with severe podocyte injury and structural abnormalities identified during the disease course. Importantly, the predominantly non-albumin proteinuria profile preceded clinical deterioration and the subsequent demonstration of advanced structural abnormalities on repeat biopsy. In this patient, lack of response to corticosteroids, rituximab, and targeted-release budesonide occurred in the setting of progressive kidney dysfunction and structural abnormalities on repeat biopsy.

In Case 3, the very low uAPR values indicate that albumin represented only a small proportion of total urinary protein. However, because urinary protein electrophoresis, immunofixation, and low-molecular-weight protein measurements were not available, the composition and origin of the non-albumin fraction cannot be established. Therefore, low uAPR should not be interpreted as direct evidence of advanced glomerular structural injury. Nevertheless, in this patient, predominantly non-albumin proteinuria was already present at initial presentation and preceded progressive kidney dysfunction and the subsequent demonstration of more advanced structural abnormalities on repeat biopsy. This temporal association is hypothesis-generating and warrants further investigation using direct urinary protein characterization.

In the second case presenting the structural transformation, uAPR remained relatively stable (0.57 and 0.63) despite progressive histological transformation toward secondary FSGS. This observation suggests that structural remodeling and podocyte loss may continue despite only modest changes in urinary protein composition. These findings support further evaluation of serial uAPR measurements as a potential tool for monitoring the dynamic evolution of glomerular injury.

Although electron microscopy remains the gold standard, it is invasive and not suitable for repeated assessments. Serial uAPR measurements may provide complementary longitudinal information between biopsies; however, whether they reflect specific ultrastructural changes remains to be established prospectively. Current understanding of IgAN pathogenesis is based on the “four-hit” hypothesis, involving aberrant glycosylation of IgA1, production of anti-glycan antibodies, formation of immune complexes, and their deposition in the mesangium [1]. However, whether the MCD-IgAN variant shares this immunopathogenic pathway remains unclear.

A study by Huixian et al. [10] investigated multiple factors potentially involved in the pathogenesis of MCD-IgAN, including levels of Gd-IgA1, anti-glycan antibodies, and mesangial inflammatory responses. Their findings suggest that MCD-IgAN represents a dual glomerulopathy, consisting of mild IgAN with superimposed MCD, which may explain its distinct clinical behavior.

Although these biomarkers were not assessed in our patients, further studies correlating uAPR with immunological markers and treatment outcomes are warranted. Given that repeat kidney biopsies are rarely performed in primary IgAN, there is a critical need for simple, cost-effective tools to monitor disease activity and prognosis. uAPR may represent such a parameter. Although these findings suggest that serial uAPR assessment may provide additional insight into disease evolution and treatment response, its clinical implementation will require prospective evaluation of analytical reproducibility, intra-individual biological variability, and clinically meaningful reference change values using standardized first-morning urine sampling.

In the era of emerging targeted therapies for IgA nephropathy, including endothelin receptor antagonists, complement inhibitors, and novel immunomodulatory strategies, improved phenotypic characterization of patients is becoming increasingly important. Although preliminary, our observations suggest that urinary protein composition assessed by uAPR may contribute to identifying distinct biological phenotypes and, in the future, may help refine therapeutic decision-making.

In clinical practice, uAPR should not be used as a stand-alone marker to guide treatment. Rather, discordance between urinary protein composition, clinical presentation, and histopathological findings may prompt closer reassessment, including consideration of additional urinary protein characterization or, when clinically justified, repeat kidney biopsy.

## 5. Limitations

This study has several limitations. It represents a small case series, limiting generalizability. uAPR was not associated with a broad panel of molecular biomarkers. Additionally, repeat biopsies were not performed systematically in all patients, and selection bias cannot be excluded. Future studies should further evaluate uAPR in larger, prospective cohorts and assess its integration with molecular biomarkers.

Another important limitation is that uAPR was calculated from single spot urine samples obtained during routine clinical care. As with other urinary biomarkers, uAPR may be influenced by biological variability, including sample timing, hydration status, edema, and ongoing therapy. Future prospective studies should therefore evaluate the reproducibility and clinical utility of uAPR using standardized serial first-morning urine samples collected under predefined conditions.

An additional limitation is the absence of a direct comparison between uAPR and qualitative urinary protein profiling. Future studies should validate uAPR against urinary protein electrophoresis, molecular-weight-based protein separation, or quantitative measurement of individual urinary protein fractions to determine how accurately it reflects urinary protein composition and the underlying pattern of glomerular barrier injury.

The heterogeneity of therapeutic approaches also limits the ability to distinguish phenotype-related from treatment-related effects.

An additional limitation is the potential influence of concomitant therapies, including RAAS blockade, corticosteroids, calcineurin inhibitors and targeted-release budesonide, on the composition of urinary protein excretion and uAPR values. Although baseline measurements were obtained before initiation of immunosuppressive therapy, treatment-related changes during follow-up may have affected longitudinal uAPR measurements and should be considered when interpreting the findings.

An additional limitation is the absence of direct characterization of urinary protein composition by electrophoresis, immunofixation, or measurement of low-molecular-weight proteins. Consequently, particularly in Case 3, the origin of the substantial non-albumin urinary protein fraction cannot be determined, and low uAPR cannot be considered specific for glomerular structural injury.

Representative histopathological and electron microscopy images from the archival biopsies were not available for inclusion in the present report, which limits direct visual assessment of the morphological findings described in the pathology reports.

Dietary protein intake was not standardized, and transient clinical factors, including intercurrent infections, particularly in Case 1, may also have influenced urinary protein excretion.

In addition, the reproducibility and biological variability of uAPR were not assessed in the present study and should be addressed in future prospective investigations.

## 6. Conclusions

In conclusion, this three-case series illustrates that urinary protein composition assessed by uAPR may provide complementary information to the clinicopathological assessment of IgA nephropathy. Although exploratory, our observations show that differences in urinary protein composition occurred alongside distinct patterns of podocyte injury and disease phenotype. uAPR should always be interpreted in conjunction with the complete clinical and histopathological picture. Further prospective studies are warranted to determine whether uAPR may serve as a useful adjunct for risk stratification and longitudinal monitoring in IgAN.

## Figures and Tables

**Table 1:** Laboratory characteristics, paired uACR/uPCR measurements, and histopathological findings at the respective kidney biopsy time points.

Parameter	Case 1	Case 2 (Baseline)	Case 2 (Follow-Up)	Case 3 (Baseline)	Case 3 (Follow-Up)
**Serum albumin [g/L]** **(35–52)**	12.7	41	37.5	33.2	36
**Total protein [g/L]** **(66–83)**	40.7	64.3	65.6	58.1	58.9
**Serum creatinine [μmol/L]** **(45–90)**	51.2	128	132	96.1	187.3
**eGFR [mL/min/1.73 m^2^]**	123.5	41	40	98.1	43.2
**Total CH [mmol/L]** **(3.0–5.0)**	13.91	8.9	9.2	10.09	10.24
**HDL CH [mmol/L]** **(>1.0)**	1.91	1.56	1.78	2.39	2.12
**LDL CH [mmol/L]** **(<3.0)**	8.63	7.6	6.0	6.36	6.1
**TG [mmol/L]** **(<1.7)**	1.23	2.45	3.61	2.19	5.72
**uACR [mg/g]**	3951.3	1532	2211	652.3	989.4
**uPCR [mg/g]**	5192	2686	3505	9600	7900
**uAPR**	0.76	0.57	0.63	0.07	0.13
**SBP [mmHg]**	102	126	132	119	132
**DBP [mmHg]**	76	78	76	65	76
**Histopathology**	IgA nephropathy (M1 E0 S0 T0 C0)	IgA nephropathy (M1 E0 S0 T0 C1)	FSGS	MCD	IgA nephropathy (M1 E0 S1 T2 C0)

CH—cholesterol; DBP—diastolic blood pressure; eGFR—estimated glomerular filtration rate, HDL-CH—high-density lipoprotein cholesterol; LDL-CH—low-density lipoprotein cholesterol; SBP—systolic blood pressure; uACR—urine albumin-to-creatinine ratio; uAPR—urine albumin–to-protein ratio; uPCR—urine protein-to-creatinine ratio.

**Table 2:** Comparison of three cases of IgA nephropathy.

Feature	Case 1	Case 2	Case 3
**Final diagnosis**	IgA nephropathy (MCD-like)	IgA nephropathy with secondary FSGS	IgA nephropathy
**Initial presentation**	Nephrotic syndrome resembling MCD	Chronic proteinuria	“MCD” on biopsy ⟶ nephrotic syndrome
**Proteinuria profile (uAPR)**	uAPR = 0.76 (albumin-predominant proteinuria)	uAPR = 0.57–0.63 (mixed proteinuria pattern)	uAPR = 0.07–0.13 (predominantly non-albumin proteinuria)
**Electron microscopy**	Diffuse foot process effacement; no GBM abnormalities	Baseline: no podocyte abnormalities; follow-up: diffuse foot process effacement with thickened and irregularly folded GBM	Diffuse foot process effacement with sparse mesangial electron-dense deposits
**Proposed dominant mechanism**	Podocyte injury	Structural damage + podocyte loss	Podocyte injury with structural abnormalities
**Disease course**	Steroid-dependent remission	Stable renal function with histological progression	Progressive renal impairment
**Treatment response**	Good	Stable kidney function despite histological progression	No response (steroids, cyclosporine, rituximab, budesonide)
**Proposed pathophysiological interpretation**	IgA podocytopathy	IgAN progressing to FSGS	Possible early IgAN not captured in the initial biopsy
**Key insight**	IgAN may mimic MCD	IgAN may progress to FSGS	IgAN may be missed in early biopsy

MCD—Minimal Change Disease; FSGS—Focal Segmental Glomerulosclerosis; IgAN—IgA nephropathy.

## Data Availability

The data presented in this study are available from the corresponding authors upon reasonable request. The data are not publicly available due to patient privacy and ethical restrictions.
